# Integrating mental health and cardiovascular wellness: synergistic impacts and the promise of comprehensive care models

**DOI:** 10.1097/MS9.0000000000003391

**Published:** 2025-05-29

**Authors:** Ajeet Singh, Rumaisa Riaz, Amogh Verma, Hamza Irfan, Ayesha Shaukat, Abdullah Nadeem, Priya Goyal, Ashna Habib, Prakasini Satapathy

**Affiliations:** a Department of Internal Medicine, Dow University of Health Sciences, Karachi, Pakistan; b Department of Internal Medicine, Rama Medical College Hospital and Research Center, Hapur, India; c Department of Medicine, Shaikh Khalifa Bin Zayed Al Nahyan Medical and Dental College, Lahore, Pakistan; d Department of Medicine, Dow University of Health Sciences, Karachi, Pakistan; e Department of Internal Medicine, Dayanand Medical College and Hospital, Ludhiana, Punjab, India; f Development of University Center for Research and, Chandigarh University, Mohali, Punjab, India; g Department of Medical Laboratories Technique, AL-Mustaqbal University, Hillah, Babil, Iraq

**Keywords:** cardiovascular disease, depression, integrated care, mental health, telehealth

## Abstract

This review explores the bidirectional relationship between mental health disorders and cardiovascular disease (CVD), highlighting the potential of integrated healthcare models to improve outcomes. While CVD remains the leading cause of global mortality, traditionally linked to risk factors like hypertension and diabetes, emerging evidence shows that mental health conditions, especially depression and anxiety, significantly increase CVD risk through mechanisms such as chronic stress, inflammation, and neuroendocrine dysregulation. Activation of the hypothalamic-pituitary-adrenal axis and sympathetic nervous system exacerbates inflammation, elevates blood pressure, and contributes to cardiovascular risk factors. Moreover, the psychological burden of CVD often worsens mental health, creating a vicious cycle that complicates treatment adherence and patient management. Integrated care models offer a holistic approach to address these interconnected issues, potentially improving clinical outcomes, reducing healthcare costs, and enhancing patient adherence. This review also explores the role of telehealth and digital health interventions in overcoming accessibility barriers, particularly for underserved populations. Finally, policy recommendations emphasize the need for increased funding, professional training in interdisciplinary care, and targeted outreach to ensure equitable access to integrated care. By addressing both CVD and mental health challenges, these models could improve quality of life and reduce the global burden of these intertwined diseases.

**HIGHLIGHTS**

- Mental health disorders, such as depression and anxiety, not only increase the risk of cardiovascular diseases (CVD) but are also more prevalent in individuals with preexisting CVD, creating a harmful feedback loop that worsens both conditions.
- Chronic stress, inflammation, and dysregulation of the hypothalamic-pituitary-adrenal axis are key biological factors linking mental health and CVD, contributing to hypertension, atherosclerosis, and other cardiovascular issues.
- Addressing mental health and cardiovascular health together through integrated care models has shown potential for improving patient outcomes, yet significant knowledge gaps remain in implementing and optimizing these approaches effectively.
- Studies consistently demonstrate the higher prevalence of mental health disorders in CVD patients, and vice versa, with clinical evidence suggesting that treating mental health conditions can improve cardiovascular outcomes, highlighting the need for comprehensive treatment strategies.

## Introduction

Cardiovascular diseases (CVDs) are the foremost contributors to morbidity and mortality worldwide. Data from the Global Burden of Disease indicates that, in 2021 alone, these conditions were responsible for 20.5 million deaths, accounting for nearly one-third of all deaths globally^[1]^. CVD includes diseases such as coronary artery disease (CAD), heart attack, and strokes, and they are still the number one cause of death globally. Even though the part played by conventional risk factors, including hypertension, diabetes, and obesity, has been thoroughly researched, it is now realized that they are not sufficient to explain the high incidence of CVD. Promising evidence currently exists on the bi-directional significant associations of mental health disorders with cardiovascular health, which indicates the interconnectedness of these health domains that have not to date been effectively captured by integrated models of medicine^[2]^. This review aims to explore this interconnection and assess how integrated healthcare models can enhance patient outcomes by addressing both conditions simultaneously.

Although research has established the association between mental health and CVD, a significant gap remains in understanding the most effective strategies for managing both conditions in a unified manner. Current healthcare approaches often treat these issues separately, leading to fragmented care and suboptimal health outcomes. The absence of standardized, multidisciplinary interventions highlights the need for research that consolidates findings from both fields to propose holistic treatment strategies. Identifying and addressing this research gap is crucial to developing evidence-based interventions that integrate mental health support into cardiovascular treatment protocols.

Depression and anxiety disorders are already recognized as essential factors that have shifted from the role of “less threats” to the leading global burdens of disease^[3]^. Depression is a universal condition that affects more than 280 million people globally, while anxiety disorders, which are also common among the population, affect millions of people regardless of their age, gender, or economic status, for instance. These mental health issues are not only psychological but are evident in other forms that directly affect the biological processes that are areas of cardiovascular systems^[4]^. For instance, depression is associated with raised inflammation and disturbances of the hypothalamic-pituitary-adrenal (HPA) axis, both of which are detrimental to CVD. Patients with anxiety disorders have a constantly stimulated sympathetic nervous system, which results in high blood pressure, increased pulse rate, and high stress hormone levels – all of which fall under the category CVD risks. The relationship between mental and CVDs has gone from a hypothetical connection to a well-developed research area since it has been demonstrated how these diseases can enhance each other^[5]^.

Adding complexity to this relationship is the recognition that the link between mental health and CVD is bidirectional^[6]^. Patients with preexisting CVD are commonly privy to heightened prevalence rates of mental health disorders caused by the psychological effects of the disease, stress related to chronic illness, as well as decreases in lifestyle flexibility as a result of their disease^[5]^. This creates a feedback loop: mental illness increases CVD risk, and CVD, conversely, increases the likelihood of poor mental health. This cycle, as described, damages not only patient outcomes but also hurts overall health systems and economies across the globe. For example, the following patients are likely to display challenges in following prescribed treatment plans, managing their diet, and gaining access to medical care to reverse sickness and overall health deterioration: patients with dual mental and cardiovascular disorders^[2]^.

Recent literature points to the view that integrated care models are viable while at the same time establishing that significant knowledge deficits persist in this field. Thus, more extensive research efforts are necessary to refine procedures, define effective practices, and introduce such models successfully into various healthcare environments.

This review contributes uniquely by synthesizing insights from cardiology, psychiatry, and pharmacology to propose an integrated care model. By analyzing key interactions between mental health and CVD, this research provides actionable insights that may guide future clinical guidelines and healthcare policy reforms. Unlike prior research that primarily examines these conditions in isolation, this review emphasizes a holistic perspective, demonstrating the necessity of a collaborative and interdisciplinary treatment framework. By addressing these critical gaps, this research aims to improve long-term patient outcomes through a more unified and systematic healthcare approach.

## The intersection of mental health and cardiovascular health

### Biological mechanisms

#### Stress response and cardiovascular health

##### Role of the HPA axis

**Figure 1.**
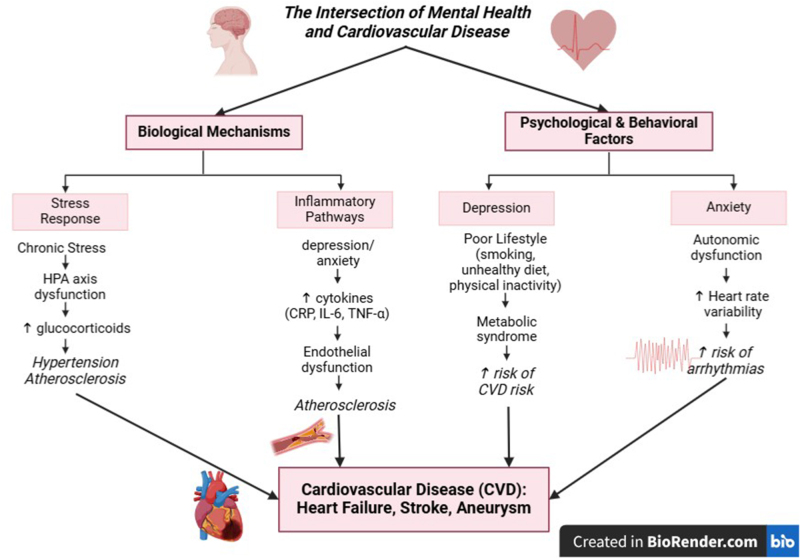
Mental health disorders as risk factors for cardiovascular disease.

The HPA axis is a complex network of neuroendocrine channels and feedback loops that regulate physiological homeostasis. It is well documented that humans and animals cope with stressors to their welfare by activating neurons that regulate neuroendocrine and autonomic responses^[7]^. The adrenal cortex secretes glucocorticoids, an identifiable feature of the endocrine response for the HPA axis. To maintain homeostasis, circulating glucocorticoids act on different kinds of tissues by limiting reproduction, inducing lipolysis and proteolysis, amplifying sympathetic nervous system-driven vasoconstriction, and changing stress-related behaviors^[8]^. Most acute stress responses, such as enhanced metabolism, cognition, and immune suppression, provide a survival advantage. However, prolonged or repeated stress exposure can lead to dysregulation of the HPA axis, resulting in chronically elevated cortisol levels that contribute to metabolic syndrome, insulin resistance, and central obesity – all of which are key risk factors for CVD^[9]^. Chronic activation of the HPA axis also induces glucocorticoid resistance in immune cells, impairing their ability to regulate inflammatory responses, thereby promoting low-grade systemic inflammation^[9]^. This persistent elevation in circulating glucocorticoids is associated with systemic inflammation, oxidative stress, endothelial dysfunction, and increased risk of metabolic disorders such as insulin resistance, obesity, and hypertension, all of which contribute to CVD^[9]^. Dysregulation of the HPA axis has physiological knock-on effects that eventually increase the risk of immune system problems, depression, metabolic diseases, and CVDs such as cardiac hypertrophy and vascular damage^[9]^ (Fig. 1).

HPA axis, hypothalamic-pituitary-adrenal; CRP, C-reactive protein; IL-6, interleukin-6; TNF-α, tumor necrosis factor alpha; CVD, cardiovascular disease.

##### Impact of chronic stress on cardiovascular risk factors

Prolonged stress exposure has been linked to hypertension, atherosclerosis, and other cardiovascular disorders. Prolonged stress causes the sympathetic adrenal medulla to become more active, lowers the hypothalamus-pituitary-adrenal axis activity, decreases anti-inflammatory capacity, and raises blood catecholamine levels^[10]^. By binding to the β-adrenal receptor on macrophage surfaces, catecholamine encourages the production of additional catecholamine by macrophages, triggering a cascade of inflammatory responses and accelerating atherosclerotic plaque formation^[11–13]^. Additionally, chronic stress promotes oxidative stress, which contributes to vascular dysfunction. Excessive production of reactive oxygen species due to prolonged stress damages endothelial cells, reduces nitric oxide bioavailability, and impairs vasodilation, ultimately leading to arterial stiffness and hypertension^[14,15]^. Over time, this imbalance predisposes individuals to hypertension, myocardial infarction, and stroke. Even if there is ongoing debate over this association, epidemiological research suggests the significance of psychological stress in the pathology of essential hypertension^[16–21]^. Research indicates that chronic stress can precipitate various cardiovascular conditions, including the dysfunction of vascular smooth muscle cells and an elevated risk of aneurysmal rupture^[22–25]^. Critically, persistent stress is a crucial factor in the initiation and progression of atherosclerosis. According to another research, Mexican women’s carotid atherosclerosis is independently associated with long-term stress^[26]^. After 2 weeks of chronic stress in the rat model of atherosclerotic intimal hyperplasia, atherosclerotic lesions obstruct most of the carotid arteries^[27]^. A large cohort study of over 10 000 people discovered that one or more degrees of psychological stress were considerably higher in myocardial infarction patients^[28]^. The overwhelming data from both human and animal studies emphasizes chronic stress’s importance in enhancing CVD advancement, particularly atherosclerosis.

#### Inflammatory pathways

##### Influence of mental health disorders on systemic inflammation

The relationship between inflammation and depression is apparent^[29]^. Patients with significant depression consistently show elevations in immune markers linked with chronic inflammation, such as tumor necrosis factor, interleukin (IL)-1 beta, and interleukin-6 (IL-6), as well as acute phase proteins like C-reactive protein (CRP)^[30]^. Postmortem brain samples from depressed people additionally demonstrate increased inflammatory responses, including microglia activation, immune cell trafficking to the brain, and activation of inflammatory signaling pathways in the brain parenchyma^[30]^. Furthermore, the administration of inflammatory stimuli like endotoxin and the typhoid vaccine, as well as inflammatory cytokines like interferon-alpha, can cause depressed symptoms. The emergence of depressive disorders is predicted by inflammatory indicators such as CRP and IL-6^[30]^. Lastly, suppression of inflammatory cytokines has been demonstrated to lessen depressed symptoms, particularly in individuals with inflammatory and autoimmune diseases^[31,32]^. It has been demonstrated that inhibiting inflammatory cytokines might lessen depressed symptoms, particularly in those with inflammatory and autoimmune diseases^[31,32]^ (Fig. 1).

##### Connection between inflammation and CVD

Systemic and localized inflammatory processes are essential factors in the onset and progression of CVD, affecting stages from endothelial dysfunction to the presentation of clinical symptoms^[33–36]^. It has been demonstrated that inflammatory biomarkers can predict CVD without referencing typical risk factors^[37–40]^. A common mechanism connecting preexisting and emerging cardiovascular risk factors to the development of atherosclerosis, which causes cerebral aneurysms, major artery thrombotic stroke, and CAD, is inflammation^[41–45]^. All stages of atherosclerosis, from atherogenic lipoprotein retention within the arterial wall to plaque development and rupture, involve a complex network of both innate and adaptive immune systems, including the bone marrow and spleen, which modulate the pro-inflammatory and anti-inflammatory responses of protein mediators such as cytokines and immune cells such as leukocytes, macrophages, and lymphocytes^[46]^. Sympathetic activation caused by mental stress increases the risk of heart failure (HF), accelerates the development of atherosclerosis, and destabilizes plaque^[47–52]^.

The sympathetic nervous system’s activity also induces platelet activation in response to mental stress. Elevated coagulability and a prothrombotic condition are caused by activated platelets releasing inflammatory cytokines and adhesive molecules that cause leukocyte recruitment on the endothelium surface. Consequently, these circumstances facilitate the attraction of leukocytes and platelets to the vessel wall, thereby encouraging the onset of arterial thrombosis^[5,48,53]^. Hence, the onset and progression of CVD are influenced by inflammation and sympathetic activity, underscoring the complex relationship among stress, immunological responses, and cardiovascular risk.

#### Neuroimmune and neurovascular interactions

Imbalances in the brain’s immune response associated with mental health disorders contribute to persistent neuroinflammation, which negatively impacts vascular function. Stress plays a crucial role in the complex interplay between the neuroendocrine and immune systems, leading to adverse physiological effects. Psychological distress activates the HPA axis and the sympathetic nervous system, initiating inflammatory pathways that elevate cardiovascular risk. Furthermore, stress-induced immune system changes promote chronic low-grade inflammation, a factor implicated in both psychiatric disorders and CVDs. Increased levels of inflammatory markers such as IL-6 and CRP serve as indicators of CVD risk while also being strongly associated with depressive symptoms, highlighting a shared underlying mechanism^[53]^.

##### Amygdala overactivity and arterial inflammation

Excessive activation of the amygdala, frequently seen in individuals with chronic stress and anxiety disorders, is closely linked to systemic inflammation and vascular dysfunction. Heightened amygdala activity corresponds with increased arterial inflammation, accelerating the onset and progression of CVD. This overactivity prompts the bone marrow to produce pro-inflammatory immune cells, which migrate into blood vessels and contribute to atherosclerosis. The inflammatory response triggered by chronic stress serves as a critical link between mental health disorders and CVD progression^[5]^.

### Psychological and behavioral factors

#### Depression and cardiovascular risk

##### Prevalence of depression in cardiovascular patients

Depression affects around 20% of individuals with CAD and may increase to 30%–40% in those with HF^[54–59]^. Over the past few decades, there has been a twofold increase in the prevalence of depression among patients with cardiovascular disorders^[60]^. Moreover, patients with myocardial infarction or HF had a threefold increased incidence of depression compared to the general population^[61,62]^.

##### Depression’s impact on lifestyle choices and adherence to treatment

Patients with CVD and depression have been shown to have poor quality of life in developing countries^[63,64]^. Patients’ work, social lives, and lifestyle choices are all impacted by depression. It could also affect their productivity, adding to the stress on their families^[64]^, resulting in hospital readmissions and raising the mortality rate from coronary heart disease (CHD)^[65,66]^. Patients with depression are less likely to adhere to healthy lifestyle habits and self-care regimens^[66]^, which increases their risk of developing CAD. Additionally, several research studies have shown that depressed patients adhere to medication and lifestyle modifications less well, which harms their medical conditions^[67–69]^.

Additionally, depression has been linked to higher rates of smoking and lower likelihood of cessation^[70,71]^, higher rates of physical inactivity^[69–71]^, higher rates of alcohol consumption^[70]^, and higher rates of dietary fat intake^[71]^. On the other hand, because of their increased physical activity, individuals with CAD who exhibit positive effects have more excellent survival rates^[72,73]^. Moreover, a patient’s adherence to healthy living practices is gradually linked to increased positive impact^[73]^ (Fig. 1).

#### Anxiety and cardiovascular outcomes

##### Relationship between anxiety disorders and cardiovascular events

Anxiety disorders are among the most prevalent psychological disorders, with severe symptoms, a high clinical burden, and challenging treatment options^[74]^. Studies have indicated a strong correlation between anxiety disorders and heart disease^[75]^. In addition, anxiety disorders themselves are a separate risk factor for death and morbidity related to CVD^[76,77]^. The patient’s general health might decline because of the interaction between these two disorders^[78]^. Thus, to inform preventative and therapeutic approaches, it is imperative to investigate the relationship between anxiety disorders and CVD.

##### Anxiety’s effect on heart rate variability and overall cardiac function

Numerous studies have repeatedly shown that people with anxiety have higher levels of inflammatory markers like homocysteine, CRP, and IL^[79,80]^. As a result, anxiety disorders can cause long-term inflammation, which can damage blood vessel endothelial linings and increase the risk of heart attacks and strokes^[81,82]^. Anxiety affects the cardiovascular system through a complicated autonomic circuit that includes the autonomic nerve system and anxiety-related nuclei^[83]^. Anxiety disorders can cause psychological stress, which can activate the HPA axis and the sympathetic nervous system. This can lead to elevated levels of cortisol, catecholamines, and other hormones, which may degrade atherosclerotic plaques and ultimately cause thrombosis, which is the primary root cause of acute coronary syndromes^[84–86]^. Moreover, anxiety disorders may increase the risk of HF and unfavorable consequences by limiting the cardioprotective effects of B-type natriuretic peptide mediated by the vagus^[87]^. All these results lead to a likely relationship between anxiety disorders and heart disease (Fig. 1).

## Evidence of synergistic impacts

### Epidemiological evidence

#### Co-occurrence of mental health disorders and CVD

For those with severe mental illnesses (SMI), CVD is the leading cause of death, just like it is in the general population^[88–90]^. The most thorough meta-analysis of CVD risk in individuals with SMI to date, which included 113 383 368 controls and 3 211 768 patients, found that SMI patients had a statistically significant higher risk of CHD overall compared to controls (54% higher risk in longitudinal studies and 51% higher risk in cross-sectional studies)^[91]^. Patients with congestive HF experience depression at a much higher rate than those in the general population^[92]^. Up to one-third of CHD patients have heightened depressive symptoms, and over one-fifth of all patients have depression (the risk of depression is highest in the most severe CHD cases). These are at least four times higher prevalence than those found in the population^[93–95]^. At the same time, depression is more common in women than in men with established congestive HF; depression in males is more strongly associated with a worse prognosis for the heart^[96]^. A meta-analysis of 30 prospective cohort studies (*N* = 893 850) found that there is a statistically significant 30% (relative risk [RR] = 1.30, 95% CI: 1.22–1.40) increase in the risk of CHD among those who have depression as opposed to those who do not^[97]^. In comparison to nondepressed individuals, depression was linked to a 31% greater likelihood of the risk of MI and a 36% increase in the risk of cardiovascular mortality, according to another meta-analysis of prospective cohort studies (*N* = 323 709)^[98]^. Despite not finding a higher risk of CHD in those with bipolar disorder (BPD)^[91]^, the most thorough meta-analysis of CVD risk in people with SMI did discover a substantial correlation between BPD and CVD in longitudinal studies. In the past 10 years, three meta-analyses^[65,88,89]^ have supported the findings of previous research indicating a possible link between anxiety disorders and incident CHD. One of them shows that anxiety is linked to a 41% increased risk of CHD^[76]^. Comparatively, to general/unspecified anxiety, phobic anxiety had a stronger correlation with incident CHD. Independent of demographics, biological risk factors, and health behaviors, another meta-analysis of 20 studies (*N* = 249 846) examining the relationship between anxiety (i.e. anxiety, panic, phobia, and anxiety) and incident CHD found that at first, healthy individuals with high anxiety had a 26% higher risk for incident CHD (hazard ratio [HR] = 1.26, 95% CI: 1.15–1.38, *P* > 0.0001)^[99]^. Depending on the diagnostic technique used, the prevalence of Post Traumatic Stress Disorder caused by heart disease varies greatly, ranging from 0% to 38%, with an average of 4%–16%, according to a recent systematic review that included 150 research^[100]^. A large-scale Swedish study (*N* = l 107 524) revealed that there is a correlation between mental illness and CHD for a variety of mental disorders, such as personality disorders, alcohol-related disorders, adjustment disorders, and other substance use disorders, with increased CHD risks ranging from 35% to 92% when compared to individuals who did not receive a diagnosis for the disorder in question^[101]^. Shen *et al*^[102]^ stated the crude incidence rate of CVD as 9.7, 7.4, and 7.0 per 1000 person-years among patients with psychiatric illnesses, their unaffected siblings, and the matched reference group throughout up to 30 years of follow-up of the Swedish cohort. In comparison to their siblings, patients with psychiatric illnesses had higher rates of CVD during the first year following diagnosis (HR, 1.88; 95% confidence interval [CI], 1.79–1.98) and beyond that (1.37; 95% CI, 1.34–1.39). This was according to longitudinal matched cohort research. Comparing the rate increases with the matched reference population revealed similar trends^[102]^. Because of the firm and conclusive link between mental health issues and CVD highlighted by epidemiological data, these susceptible individuals must receive integrated treatment approaches that address their mental and cardiovascular health.

### Clinical evidence

#### Impact of mental health treatment on cardiovascular outcomes

Numerous research studies have investigated how treating anxiety and depression affects cardiovascular outcomes (Table 1). Wu *et al*^[103]^ observed 7419 post-MI patients from a Taiwanese registry and discovered a j-shaped distribution of outcomes: patients on low doses of benzodiazepines experienced reduced rates of hospitalizations for HF, cardiovascular mortality, and sudden death; however, at higher doses, the benzodiazepines lost their protective effects and were linked to an increased risk of sudden death. Patients treated with benzodiazepines as opposed to non-benzodiazepine Z-drugs showed a trend toward increased mortality and a greater likelihood of rehospitalization in a trial of HF patients with insomnia^[104]^. To better understand the effects of benzodiazepines on the HF population and the impacts of anxiety on HF patients, more research is required. According to a study by Carmin *et al*^[105]^, receiving mental health treatment for depression or anxiety – whether through medication or psychotherapy – was linked to a notable and significant decrease in the risk of either an emergency department (ED) evaluation (74% risk reduction if receiving both treatments, 49% for medication treatment alone, and 53% for psychotherapy alone) or a being readmitted to the hospital (75% risk reduction if receiving both treatments, 49% for psychotherapy alone, and 58% for medication alone). According to these results, mental health therapies are crucial for lowering ED visits and hospital stays in patients with concomitant anxiety or depression and HF ^[105,106]^. Some studies have evaluated secondary endpoints associated with the disease outcome. For example, SADHART-CHF (Sertraline Against Depression and Heart Disease in Chronic HF)^[107,108]^ found a trend toward fewer hospitalizations for HF patients receiving depression treatment. The exact processes via which mental health interventions are linked to decreased rates of ED visits, hospitalizations, and overall mortality are still unknown. Nonetheless, these results align with the present comprehension of the heart-brain link.

**Table 1:** Key studies supporting the findings on mental health and cardiovascular disease

Study	Main findings	Results	Key interactions
Wu *et al*, 2014	Moderate doses of anti-anxiety medication improve cardiovascular outcomes, while higher doses increase risks.^104^	A J-curve effect was observed: low to moderate benzodiazepine (BZD) doses reduced cardiac mortality and heart failure hospitalization, but higher doses (>5 mg/day) increased sudden death risk.^104^	Mental health management can positively influence cardiovascular outcomes, highlighting the need for integrated care models.^104^
Sato *et al*, 2020	Benzodiazepine use in heart failure patients with insomnia increases rehospitalization risk compared to Z-drugs.^105^	Patients on benzodiazepines had a 1.5-fold higher risk of rehospitalization for heart failure than those using Z-drugs.^105^	Optimizing insomnia treatment in heart failure patients may help reduce hospital readmissions.^105^
Carmin *et al*, 2024	Mental health treatment significantly reduces hospital readmissions and mortality in heart failure and ischemic heart disease patients.^106^	Patients receiving both psychotherapy and antidepressants had 75% fewer rehospitalizations, 74% fewer emergency visits, and 66% lower mortality.^106^	Integrating mental health treatment into cardiovascular care improves patient outcomes.^106^
Jiang *et al*, 2011	Depression remission in heart failure patients is associated with fewer cardiovascular events and better survival.^108^	Patients whose depression remitted had significantly lower cardiovascular events and longer survival than those whose depression persisted.^108^	Treating depression in heart failure patients may improve cardiovascular prognosis.^108^
Rotvig *et al*, 2022	Cardiac drug therapies, especially beta-blockers and antiarrhythmics, are linked to increased anxiety in cardiac patients.^123^	Patients using beta-blockers, antiarrhythmics, and diuretics had significantly higher self-reported anxiety symptoms.^123^	Neuropsychiatric side effects of cardiac drugs should be considered in managing mental health and cardiovascular disease together.^123^
Zhang *et al*, 2022	Certain cardiovascular drugs, including diuretics and calcium channel blockers, increase depression and anxiety risk.^122^	Diuretics, nitrate esters, and calcium channel blockers were associated with higher depression and anxiety risks, while aspirin and statins had protective effects.^122^	Mental health assessments should be integrated into long-term cardiovascular medication management.^122^
Thompson *et al*, 2014	Anxiety and depression worsen symptom perception in atrial fibrillation patients.^117^	Increased anxiety and depression worsened atrial fibrillation symptoms, even after treatment with catheter ablation or antiarrhythmic drugs.^117^	Psychological interventions may improve overall symptom relief in AF patients.^117^
Huffman and Stern, 2007	Cardiovascular medications can cause neuropsychiatric side effects, including depression and anxiety.^110^	Beta-blockers, diuretics, and ACE inhibitors have been linked to increased depression and fatigue, while calcium channel blockers and propranolol have potential anxiolytic benefits.^110^	Careful selection of medication is crucial to balance cardiovascular benefits with mental health risks.^110^

ACE inhibitors, angiotensin-converting enzyme inhibitors; AF, atrial fibrillation; BZD, benzodiazepine.

#### Impact of cardiovascular treatments on mental health

Beta-blockers, lipid-lowering medications, angiotensin-converting enzyme (ACE) inhibitors, diuretics, and antiarrhythmics are frequently recommended to patients with heart conditions to address their underlying cardiac conditions. There is continuous discussion about the potential drawbacks of these treatments^[109]^, even with their well-established advantages, which include increased survival rates in patients with HF^[110]^, myocardial infarction, and arrhythmias^[111]^. For example, poorer psychological functioning is believed to be linked to the process by which lipophilic beta-blockers, such as metoprolol, damage the blood-brain barrier^[112]^. Moreover, 15% of individuals on the antiarrhythmic medication amiodarone have been found to have thyroid abnormalities, which can result in mood, cognitive, and psychotic symptoms^[113–115]^. The present number of primary research examining the relationship between cardiac medication therapy and the likelihood of experiencing anxiety symptoms is contradictory and founded on limited sample sizes^[116–120]^. According to a meta-analysis, individuals with CVD should have their use of cardiac medications considered when assessing their anxiety and depression^[121]^. In the Rotvig *et al*^[122]^ study, 2891 (32%) of the 8998 respondents reported having anxiety symptoms. Digoxin, antiarrhythmics, beta-blockers, ACE inhibitors, and angiotensin receptor antagonists have all been linked to neuropsychiatric adverse effects^[122]^. To maximize patient results, it is essential to carefully weigh the advantages of cardiac drugs against any potential mental health risks, as these effects may impact psychological well-being.

## Integrated care

Integrated care, also known as integrated health or coordinated care, is a transformative approach to healthcare, especially in psychiatric and physical health problems^[123]^. This model aims to improve patient satisfaction and clinical status by facilitating coordination between caregivers, especially for chronic diseases like depression and CVDs^[124]^. Integrating personal care is founded on recognizing that mental and physical health are closely interconnected, significantly influencing the other’s condition. For example, people with serious mental illnesses (SMIs) such as schizophrenia have more vulnerability to developing CVD, which can be negatively affected by other lifestyle choices such as excessive intake of fatty foods and a sedentary lifestyle^[125]^. Hence, implementing mental health services within primary care can enhance the identification of clinical risk factors for CVD and subsequently improve outcomes^[126]^.

There is ample evidence supporting the effectiveness of integrated healthcare models; for instance, combining the care of diabetes and depression can improve a patient’s outcomes significantly since depression is correlated to cardiovascular health^[127]^. Studies have shown that collaborative care models have been successfully implemented across different levels of care. For instance, a combination of mental health and diabetes care in residential programs has had a favorable result on people with SMIs^[128]^. Furthermore, the Veterans Affairs healthcare system has also utilized integrated care models to support and treat multiple somatoform disorders, depression, post-traumatic stress disorder, and other physical or medical conditions that veterans present with, and this has led to enhanced treatment retention and overall health^[129]^.

The evidence supporting collaborative care extends beyond individual case studies. Several systematic reviews have also demonstrated the efficacy of integrated care in managing behavioral, mental, and physical health outcomes across various populations^[130]^. For example, a published systematic review of integrated chronic care models described seven studies. It concluded that these models could increase access to care, improve treatment quality, and facilitate the management of co-morbid conditions with little or no increase in cost^[130]^.

Furthermore, interventions like the Behavioral Health Home (BHH) model have been developed to provide coordinated care for people with SMI, including cardiovascular risk reduction. The BHH model focuses on integrating behavioral health services with primary care to improve overall health outcomes. While one study demonstrated some benefits in cardiovascular health in patients with chronic diseases, the results differ from another study that has revealed little or no changes whatsoever^[131,132]^. Recent research also highlights the Health Outcomes Management and Evaluation Study, which tested an integrated behavioral health home model and showed promising results in addressing both physical and mental health needs. The model was particularly effective in improving metabolic outcomes in individuals with SMIs, though challenges remain in long-term sustainability^[131]^. Additionally, lifestyle interventions tailored for individuals with SMIs, such as those implemented in the InSHAPE program, have demonstrated benefits in reducing cardiovascular risk factors through structured physical activity and nutritional guidance^[125]^.

Furthermore, a functional medicine approach to integrated care emphasizes personalized treatment plans that address both psychological and physiological aspects of chronic illness. This approach has been shown to enhance patient engagement and long-term adherence to treatment^[123]^. Despite these advancements, barriers such as healthcare system fragmentation, financial constraints, and disparities in access continue to limit the widespread implementation of integrated care models. Addressing these challenges requires stronger policy support, increased provider training, and improved care coordination strategies.

## Patient-centered medical home and telehealth

The Patient-Centered Medical Home (PCMH) model is another model of care that has also been deemed helpful in managing chronic diseases, including mental and cardiovascular disorders. It is a new model of care that focuses on whole-person orientation, coordinated and accessible healthcare for patient needs^[133,134]^. The structure of PCMH is defined by teamwork, in which primary care providers collaborate with physicians, specialists, nurses, and other workers from different specialties. The collaborative model has been effective in increasing health standards, increasing patient satisfaction levels, and bringing down the costs of healthcare^[133]^.

Evidence shows that PCMHs can reduce hospitalization rates and ED utilization in patients with long-term diseases^[135]^. For example, a study demonstrated that elderly patients receiving PCMH services had better control of chronic diseases, improving their overall health and decreasing their healthcare utilization^[136]^. This is beneficial for the patients in that it can significantly reduce the cost and time involved in an in-person visit to a healthcare provider.

It has also become apparent that telehealth and other digital approaches constitute essential elements of chronic conditions care delivery during and after COVID-19^[137]^. Telehealth leads to equal access to all that can be done through remote consulting, hence eliminating the need for the patient to access the healthcare provider physically; this is of great advantage to patients with mobility problems or patients in areas with limited access to healthcare providers^[138]^. A systematic review, including studies using telehealth services in CVD management, showed the effectiveness of these services in enhancing results such as blood pressure regulation and reduced hospitalization rates^[139]^. Telehealth services can complement the PCMH model because it is proven to facilitate care coordination and guarantee that the patient will receive all the necessary help at the right time, consequently championing the management of chronic diseases.

Telehealth has also proven to be a viable option for mental health, where most people struggled to get access to therapy due to the COVID-19 outbreak^[140]^. It is noted that tele-psychological services are popular among the rural population because, after the reception, many patients state an increase in satisfaction and the absence of social stigma for receiving mental treatment by turning to a specialist through the internet^[141]^. Digital health also entails mobile apps and online platforms for therapy and has also been identified to increase attendance and compliance with clinical management programs for mental health illnesses^[142]^.

The efficacy of digital health tools and remote monitoring in managing chronic conditions cannot be overstated. A longitudinal study demonstrated that patients with chronic CVDs who utilized a fourth-generation telehealth program experienced lower hospitalization rates and reduced healthcare costs than those receiving traditional care^[143]^. Telehealth interventions for secondary prevention of CHD have similar outcomes to traditional rehabilitation programs. They have been shown to improve exercise capacity, reduce mortality rates, and enhance overall quality of life^[144]^. These results demonstrate the potential of telehealth to improve chronic disease management and patient outcomes.

## Challenges and future recommendations

Integrating care adds value because it focuses on delivering healthcare products through the organization and coordination of different parts of patient care delivery. However, there are significant challenges to the effective implementation of integrated care. Such barriers can be grouped as systemic/structural and patient-level barriers.” This article helps identify some of the obstacles to this process and thus informs the necessary changes to deliver efficient care to patients with CVDs.

Systemic and structural barriers primarily stem from the fragmentation of healthcare systems. Fragmentation occurs when different healthcare providers and services operate independently, leading to disjointed patient care experiences^[145]^. This lack of coordination can result in patients receiving incomplete or conflicting information about their health conditions, which is particularly detrimental for those with chronic diseases such as cardiovascular conditions^[146]^. Financial limitations add to these problems; many healthcare systems need help affording the investment required for integrated care. A national survey indicated that budget issues significantly reduce the benefits of teamwork between health professionals in these programs^[147]^. Additionally, scheduling conflicts, transportation barriers, and other logistical obstacles can limit patient access to healthcare services^[148]^. These barriers contribute to treatment delays and insufficient management, ultimately leading to poorer health outcomes.

At the patient level, barriers such as reluctance and stigma can significantly impact the effectiveness of integrated care. Many patients may feel hesitant to engage with healthcare services due to previous negative experiences or societal stigma associated with certain health conditions, including CVDs^[149]^. This reluctance can prevent patients from seeking timely care or adhering to treatment plans, which is crucial for managing chronic conditions effectively. Furthermore, stigma can deter patients from discussing their health concerns openly with providers, leading to miscommunication and inadequate care^[149]^.

Future research should address critical gaps in understanding integrated care models, particularly in their long-term impact on mental and physical health outcomes across diverse populations. Longitudinal studies are needed to evaluate how these models affect patient health over extended periods, providing insights into sustainability and effectiveness. Additionally, cost-effectiveness analyses can elucidate the financial implications of implementing integrated care, particularly those incorporating telehealth and digital interventions, demonstrating their value to policymakers and healthcare providers. Furthermore, enhancing the understanding of patient experiences and satisfaction will be crucial in refining care delivery, while investigating how integrated care can mitigate health disparities in underserved communities is vital for promoting equitable access.

Policymakers should explore several strategic steps to enhance the implementation of integrated care models. First, increased funding for collaborative care initiatives can support integrating mental and physical health services within healthcare systems. Financial incentives for healthcare providers participating in these models can encourage adoption and adherence to integrated practices. Additionally, investing in training programs for healthcare professionals on inter-professional collaboration and communication is essential to enhance care coordination. Addressing systemic barriers, such as administrative challenges and financial constraints, is necessary to create a more cohesive healthcare environment. Lastly, developing targeted outreach programs to engage communities, particularly those in rural or underserved areas, will facilitate better access to integrated care and ensure patients receive the comprehensive services they need.

## Limitations of the work

This review acknowledges some limitations. Observational studies rather than randomized controlled trials were used, restricting causal inference between mental health disorders and CVD. Differences in study methodology, population demographics, and diagnostic criteria affect the generalizability of findings. Moreover, the lack of longitudinal data prevents understanding the long-term effects of integrated care models.

Although attempts have been made to control them, sociodemographic variables such as lifestyle and socioeconomic status may still influence the effect observed. It may also be limited in its coverage of new developments related to digital interventions and telehealth. Other systemic barriers, including those related to healthcare infrastructure and finance, remain poorly understood yet could fundamentally affect research findings.

Most studies focus on specific geographical areas, limiting the generalization of these findings to subpopulations. Future research addressing these limitations will better inform the implementation of integrated healthcare approaches for mental and cardiovascular health.

## Conclusion

In conclusion, the intricate bidirectional relationship between CVD and mental health disorders demands a nuanced and integrative approach to patient care. Mental health conditions such as depression and anxiety not only exacerbate the risk of CVD through physiological pathways like inflammation and stress response but also complicate recovery and management for those already suffering from CVD. Conversely, CVD often leads to significant psychological stress, further compounding patients’ health challenges and creating a feedback loop that worsens outcomes on both fronts. The evidence highlights the significance of integrated care models that address mental health and cardiovascular conditions, demonstrating benefits in patient adherence, reduced hospitalizations, and improved health outcomes. Successfully implementing these models, however, involves tackling challenges such as disjointed healthcare systems, logistical hurdles, and societal stigma. Future research should focus on enhancing integrated care methods and exploring digital health innovations to ensure accessible and coordinated care, especially for underserved populations. These initiatives are vital for progressing toward a comprehensive, patient-centered healthcare system that recognizes the close connection between mental and cardiovascular health.

## Data Availability

Not applicable.
